# Move and Be Moved: The Effect of Moving Specific Movement Elements on the Experience of Happiness

**DOI:** 10.3389/fpsyg.2020.579518

**Published:** 2021-01-15

**Authors:** Jenneke van Geest, Rosemarie Samaritter, Susan van Hooren

**Affiliations:** ^1^Faculty of Health Care, Academy of Arts Therapies, Zuyd University of Applied Science, Heerlen, Netherlands; ^2^KenVaK Research Centre for the Arts Therapies and Psychomotricity, Heerlen, Netherlands; ^3^Department of Arts Therapies, Codarts University of the Arts, Rotterdam, Netherlands; ^4^Faculty of Psychology, Open University of the Netherlands, Heerlen, Netherlands

**Keywords:** dance movement therapy, emotion, body feedback, interaffectivity, embodiment, positive affect, Laban Movement Analysis

## Abstract

Dynamic body feedback is used in dance movement therapy (DMT), with the aim to facilitate emotional expression and a change of emotional state through movement and dance for individuals with psychosocial or psychiatric complaints. It has been demonstrated that moving in a specific way can evoke and regulate related emotions. The current study aimed to investigate the effects of executing a unique set of kinetic movement elements on an individual mover’s experience of happiness. A specific sequence consisting of movement elements that recent studies have related to the feeling of happiness was created and used in a series of conditions. To achieve a more realistic reflection of DMT practice, the study incorporated the interpersonal dimension between the dance movement therapist (DMTh) and the client, and the impact of this interbodily feedback on the emotional state of the client. This quantitative study was conducted in a within-subject design. Five male and 20 female participants (mean age = 20.72) participated in three conditions: a solo executed movement sequence, a movement sequence executed with a DMTh who attuned and mirrored the movements, and a solo executed movement sequence not associated with feelings of happiness. Participants were only informed about the movements and not the feelings that may be provoked by these movements. The effects on individuals were measured using the Positive and Negative Affect Schedule and visual analog scales. Results showed that a specific movement sequence based on movement elements associated with happiness executed with a DMTh can significantly enhance the corresponding affective state. An additional finding of this study indicated that facilitating expressed emotion through movement elements that are not associated with happiness can enhance feelings such as empowerment, pride, and determination, which are experienced as part of positive affect. The results show the impact of specific full-body movement elements on the emotional state and the support outcome of DMT on emotion regulation.

## Introduction

The expression of emotions through body movement is one of our earliest ways of communication, preceding spoken language, and the use of movement to identify, address, and support change in emotional state has since long been recognized in the research literature (Darwin, 1872; Fuchs and Koch, 2014; Shafir, 2016). Dance movement therapy (DMT) prioritizes dance and movement as an emotional experience. It uses movement as locus for expression of emotions, to foster emotional resiliency and as an agent of change within the dance therapeutic context (Fuchs and Koch, 2014; Acolin, 2016; Tsachor and Shafir, 2017; Payne et al., 2019). An important aim of DMT includes the support of the client to unfold and articulate inner sensations, impulses, and expression of emotional content. Exploration of specific elements of movement and vitalizing movement experiences can provide the client with the possibility to connect to embodied sources of joy and well-being, and have an effect on the clients’ self-esteem, improve well-being, and increase positive emotional experiences (Koch et al., 2007, 2019; Gendlin, 2012; Gordon, 2014).

Dance movement therapy and its clinical practice builds its theoretical background by relating and contributing to existing theoretical traditions that are in accord with DMT principles. Embodiment is a concept that matches the theory and practice of DMT. The term embodiment refers to theories and research in which the body and movement play an integral part and central role in our thinking, feeling, perception, and action (Laird, 1984; Strack et al., 1988; Merleau-Ponty, 2003; Winkielman et al., 2015). One of the main assumptions of embodiment research is that of bidirectionality between subjective experience (for example, an emotion) and the body’s sensations and movements contributing to this experience (Wallbott, 1982, 1990; Riskind, 1984; Zajonc and Markus, 1984; Neumann and Strack, 2000). Hence, the way in which we are moving does not only affects how others understand our non-verbal expressions (expressive function: Darwin, 1872). Following the James-Lange theory, this movement behavior also influences our emotional experience through our kinesthetic body feedback (impressive function: James, 1884; Laird, 1984; Riskind, 1984; Strack et al., 1988; Wallbott, 1990; Fuchs and Koch, 2014; Koch et al., 2014). In this feedback process, the body receives and supplies intero- and proprioceptive information (sensory data) via the central nervous system to the brain, which results in the formation of neural patterns. In turn, these play a causal role in the experience of, for example, emotions and feelings (Damasio, 2001; Bechara and Damasio, 2005). Imagine a person who is feeling sad. Their body posture and movement may be collapsed, slowed down, and directed to the ground. Based on the reciprocal influence of body- and cognitive-affective systems and circuits, this movement also reinforces the person’s feeling of sadness. This way, emotions can be regulated by the conscious use of movement and the associated body feedback.

The impact of movement and movement tendencies such as gestures and body posture has received a lot of attention in the scientific field, with inconsistent findings. Some studies demonstrated a direct influence on affect. For example, studies showed that people’s affective responses can be influenced by the proprioceptive input coming from specific body postures (Cuddy et al., 2018), specific gestures like arm extension and flexion (Cacioppo et al., 1993), and facial expression such as smiling or pouting (Strack et al., 1988; Laird et al., 1989; Duclos and Laird, 2001; Davis et al., 2010). However, a large replication study did not find a relation between people’s affective responses and their facial expression (Wagenmakers et al., 2016). The inconsistency of findings may be explained by differences between static expressions involving a limited part of the body (face/limbs) versus dynamic movements involving the whole body. The latter includes the Body parts doing the movement and their actions, the qualitative dynamics of the muscular contraction or Effort, the change in the mover’s Shape, and the advancement of the movement through Space. It seems timely to analyze the influences of dynamic whole-body movement on affective responses and to specify findings for a clinical application such as DMT, which emphasizes the dynamic character of movement.

Emotions are not only felt on the individual level due to the proprioceptive-kinesthetic feedback on one’s own affect, but are also displayed and visible in expression and behavior within our environment. According to Fuchs and Koch (2014), this also induces processes of interaffectivity, where our body is affected by the other’s expression, and we experience the kinetics and intensity of his/her emotions through our own bodily kinaesthesia and sensations. This interactional resonance implies a dynamic mutual feedback between two bodies which can occur through the visual, auditory, or tactile channel, but also through the kinesthetic channel (Koch, 2011; Koch et al., 2011). DMT actively addresses this interaffectivity. The therapeutic relationship is achieved through movement and dance (Chaiklin and Wengrower, 2015). In DMT, movement intervention occurs in a variety of relational modes showing strong similarities with embodied relationships and self-other relatedness as known from dance (Samaritter and Payne, 2013; Samaritter, 2020). For example, we distinguish a solo movement situation without a partner, from a partnered movement situation (duet), during which there is a focus on a partner (therapist or peer). During individual (solo) movement the clients’ own movement expressions directly affect themselves and form feedback for their own emotional experience (impression of their own expression), whereas in a shared movement situation, both persons simultaneously experience their own and each other’s emotions through the process of interaffectivity (Samaritter and Payne, 2013; Fuchs and Koch, 2014). In DMT, the therapist will actively route the therapeutic relationship using his/her own bodily movement to attune to the movement patterns of the client. By using the technique of modal mirroring (Eberhard-Kaechele, 2019), the therapist relates to the client in shared whole-body actions and mirrors him/her by moving in response with the same mode of emotional expression and movement elements. Through the highly attuned, mirrored movement intervention of the therapist, the client is offered a double impression: by sensing the other individual via the visual, auditory channel, while at the same time s/he is experiencing her/his own movements through the kinaesthetic senses. The client may develop a sense of being seen, and awareness about the expressed emotions and state may increase (McGarry and Russo, 2011). Schore (2003) proposed that the brain responds with a discharge of dopamine when a person is mirrored during movement engagement with as close as possible similar movement patterns, resulting in positive feelings like pleasure and bonding to each other. In order to reflect a more realistic situation of DMT practice in research, it can be meaningful to extend the bidirectionality model and to incorporate these different loops of body feedback into the research design. Therefore, in addition to dynamic body feedback, the present study also incorporates the interactive dimension that takes place between the therapist and client, and the consequences of this dynamic body feedback on affective state.

To observe, analyze, and assess movement patterns, most dance movement therapists (DMTh) use Laban movement analysis (LMA; Bartenieff and Lewis, 1980; Laban and Ullman, 1980). LMA provides a theory-based tool to look at the quality of movement in a quantifying manner, and classifies movement components in four main categories: Body (*what moves?*), Effort (*how does the body move?*), Shape (*how does the shape of the body change?)*, and Space (*where does the body move?*). LMA identifies aspects of movement with words such as strong, free, direct, forward, rising, or retreat (Studd and Cox, 2013). In DMT, the therapist uses LMA to observe and attend to the client’s movement to change and expand movement components, observe subtle expression of emotion, share these observations with the client, and suggest how to use movement components to foster emotional resiliency in everyday life (Laban and Ullman, 1980; Shafir et al., 2016; Tsachor and Shafir, 2017). In research, LMA has been used to describe movement associated with diverse topics from personality (Levy and Duke, 2003; Van Koningsveld, 2011), mental health issues (Koch et al., 2007; Samaritter and Payne, 2017), interpersonal behavior (Roudposhti et al., 2012), emotion and emotional expression (Lourens et al., 2010; Gross et al., 2012; Shafir et al., 2016), and affect, attitude, and cognition (Koch, 2014; Koch et al., 2014).

Previous studies within the DMT domain place the dynamic, qualitative components of self-movement and the effect on the emotional experience at the forefront of their research. In his study (*N* = 60), Günther (2006) compared a jump movement (rope jumping) with a kick movement (kicking the ball). The light, continuous and rhythmic quality of the jumps caused more relaxation and joyful, indulgent, peaceful, and playful feelings among the participants. The pounding and spurting quality of the kick movement caused more negative feelings, including tension, aggression, and conservatism. The results from the study by Koch (2014), focusing on the changing shape of the body, show that after performing enclosing movements of the arms and opening movement of the hands, participants felt significantly more peaceful and relaxed, while performing an avoidance movement evoked more tension and aggressive feelings. In another study by Koch et al. (2014), participants reported more positive feelings and memories after moving with a light, free, and indulgent quality, while moving with a fighting quality (powerful, strong, and bound movements) became more related to negative feelings and memories. In their study, Shafir et al. (2013) demonstrated that the dynamic whole-body movement expression of discrete emotions (happiness, fear, and sadness) also enhanced this emotion among the participants. In a follow-up study by Shafir et al. (2016), specific sets of Laban movement elements were identified which, when applied, elicit feelings of the associated emotion (happy, angry, scared, and sad). Participants (*N* = 80) applied different combinations of LMA movement elements and rated the emotion they felt while moving. Results showed that each individual discrete emotion was predicted by a unique set of LMA movement elements and that no Laban movement element was significantly predictive for more than one emotion. The opposite effect has also been demonstrated: when the movement expression/characteristics associated with a specific emotion of an individual is limited, this leads to a reduced experience of this emotion (Laird and Lacasse, 2013; Koch et al., 2014; Shafir et al., 2016).

Results from the studies mentioned above indicate that integrating specific movement elements within a movement intervention can influence emotion. In a further exploration within the DMT domain, a few studies base their intervention on Laban and Laban theories (Koch et al., 2007; Koch, 2014; Shafir, 2016; Tsachor and Shafir, 2017) or are aimed at improving mood, affect, or emotion regulation (Haboush et al., 2006; Betty, 2013; Gordon, 2014; Punkanen et al., 2014). These findings are important for the DMT domain and are useful for the development of movement interventions where emotion regulation is an important goal. However, there is little concrete research into which specific movement elements within a movement intervention actually have their effect on a particular emotion. For the evaluation of outcomes for the DMT practice, the relationship between a specific dynamic movement intervention danced solo or together with a DMTh and the experienced emotional state with the mover would therefore be particularly relevant for further investigation.

Presenting the mover with vitalizing movement interventions to experience sources of happiness and increase positive affect is a frequently utilized practice within DMT (Koch et al., 2007), as well as offering embodied experiences to recuperate emotional expressiveness (Harris, 2007). The DMTh will guide the client in expressive movement sequences to overcome emotional stagnation and to express and experience emotions such as anger, sadness, happiness, or fear. This focus is in line with the new concept of positive health, which defines health as people’s ability to adapt and self-manage in response to the physical, emotional, and social challenges of life (Huber et al., 2016, p. 1). In DMT theory and research, interventions and treatment that address the increase of positive affect are not yet reflected well (Gordon, 2014). Rather, research focuses primarily on reducing negative emotions or symptoms, assuming that this leads to an increase in positive affect. In addition to reducing negative symptoms, it is also an increasingly important focus in therapeutic practice to support clients in experiencing positive emotions (Van der Meer, 2010; Gordon, 2014). A positive emotion like happiness can serve as a protective factor and strengthen resiliency. It seems meaningful to further integrate and extend the influence of dynamic movement and investigate the resulting body feedback on an emotional state, and more specifically happiness, into research within DMT. Such research would make a unique contribution by focusing on the relation between movement and emotion, and thereby build on the theoretical framework that answers the question of how DMT works.

The aim of the present study is to investigate whether the experience of happiness can be enhanced through the use of a dynamic movement intervention based on specific LMA movement elements associated with the expression and experience of happiness (see Table 1 for specific LMA “Happy” movement elements used for this movement intervention). In this study, we aim to differentiate between performing a movement sequence with a DMTh and performing the same movement sequence alone. Including an interactive dimension taken from DMT techniques offers a more realistic reflection of the practical DMT context. Results of this study can extend existing findings in body feedback research by incorporating dynamic movement and the interbodily resonance that takes place between the therapist and client, and the consequences of this dynamic body feedback on the affective state. Following up on the study by Shafir et al. (2016), this study aims to verify and broaden previous results by including young adults (18–25 years old) with no LMA experience or training. Following the results from neuroscience, participants’ brain responses to seeing an action are influenced by their acquired motor skills (Calvo-Merino et al., 2005; Borgomaneri et al., 2015). Participants who are not trained and experienced in LMA may have a different association between movement based on these Laban movement elements and emotion. Furthermore, the present study aims to make a contribution to the body of research that ultimately can assist DMTh in helping clients to build positive affect, in support of mental health and well-being.

**TABLE 1:** Laban movement elements associated with the experience of happiness used in the study. The left column in the table shows the main categories of movement of LMA. Each category contains several factors and qualities of movement which are described in the middle column, and are identified to predict the experience of happiness, found by studies in the right column.

Body	*Body action – jump*	Günther, 2006; Koch et al., 2007; Shafir et al., 2016
Effort	*Free flow*	Montepare et al., 1999; Koch et al., 2014; Shafir et al., 2016
	
	*Light weight*	Lourens et al., 2010; Koch et al., 2014; Shafir et al., 2016

Shape	*Spreading*	Montepare et al., 1999; Gross et al., 2012; Koch, 2014; Cuddy et al., 2015; Shafir et al., 2016
	
	*Rising*	Duclos et al., 1989; Flack et al., 1999; Cuddy et al., 2015; Shafir et al., 2016

Space	*Direction – up*	Flack et al., 1999; Dael et al., 2012; Shafir et al., 2016

Phrasing	*Rhythmicity – reinitiating*	Koch et al., 2007; Dael et al., 2012; Shafir et al., 2016

We hypothesized that happiness will be enhanced the most when a participant is supported by a DMTh who uses targeted DMT techniques, including attuning and mirroring, compared to dancing the associated movement elements independently. Second, it is expected that a single dance movement intervention based on LMA movement elements associated with the expression of happiness leads to a greater increase in the experience of this emotion, compared to a single dance movement intervention that does not contain the LMA movement elements associated with the expression of happiness.

## Materials and Methods

### Participants

Twenty-five participants (5 males, 20 females; 22 Dutch residents, 3 non-Dutch residents) participated in this study. Included participants were young adults between the age of 18 and 25 (M = 20.72, SD = 2.05). Exclusion criteria for participation were (1) experiential knowledge with LMA and DMT (e.g., from specific training or working experience as a DMTh) and (2) physical limitations that would prevent candidates from performing the movements in all conditions. Participants were recruited by means of posters at locations of Zuyd University of Applied Sciences, using this university’s student mailing lists and by directly addressing these students on-site. A snowball effect was created by posting a call on social media such as Facebook and LinkedIn, so that external participants in this age group could also register for the study. We announced that the study aimed to investigate the effects of muscle activation on mood. We advertised that we raffled gift vouchers among participants as a reward and that all participants received a certificate to prove their participation in case they could use it to obtain course credits. All participants voluntarily participated in the study and gave their informed consent.

### Design

The study had a within-subject design and each participant engaged in all three conditions. In condition A, the participant performed a movement sequence based on specific LMA movement elements associated with and to enhance happiness, together with a DMTh who attuned and mirrored the movements. In condition B, the participant performed a solo movement sequence based on specific LMA movement elements associated with and to enhance happiness. In condition C, the participant performed a solo movement sequence based on specific LMA movement elements whose execution is not associated with, or to enhance happiness. Conditions were randomized to balance and counteract learning and/or fatigue effects. This led to six different orders of conditions (A-B-C, A-C-B, B-A-C, B-C-A, C-A-B, and C-B-A). Questionnaires were presented to the participants before and after executing each condition, and at the end of the study.

### Experimental Design and Procedures

#### Development of the Movements

To select the appropriate movement sequences for all three conditions of the study, a trio of movement sequences was composed. In order to decide on the movement sequence that could be used in condition A as well as B, two different and short (40 s) movement sequences were compiled. These were based on results from studies that showed LMA movement elements that are characteristic for the expression and experience of the emotion of happiness (see Table 1). In order to develop a movement sequence intended for use in condition C, another short sequence (40 s) was created which contained LMA movement elements that are *not* associated with the expression and experience of happiness, based on the results by Shafir et al. (2016). According to these results, the LMA movement elements selected for this sequence are more likely to predict the experience of emotions such as anger, sadness, or fear. All three movement sequences demanded the same amount of physical effort based on face validation and heart rate measurement in one DMTh while executing the movements. All sequences have been validated by a Certified Laban Movement Analyst (CLMA) based on the intended movement elements. This CLMA was not aware of the purpose of the study.

**FIGURE 1:**
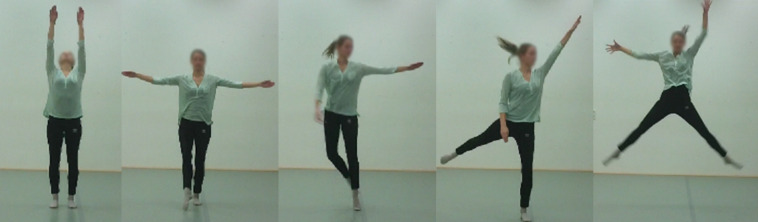
Fragments from movement sequence used in condition A and B that include a combination of LMA movement elements associated with happiness: *rising* (*Shape*), *spreading* (*Shape*), *jump left* and *upwards* (*Body action* and *Space*) combined with *free flow* (*Effort*). Photo published with permission of a volunteer.

**FIGURE 2:**
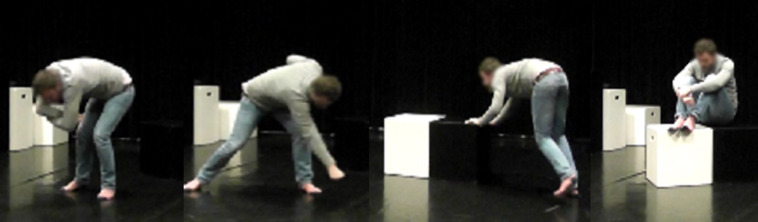
Fragments from movement sequence used in condition C that include a combination of LMA movement elements not associated with happiness: *enclosing* and *sinking* with *wringing* (*Shape* and *Effort*), *punching downward* (*Effort* and *Space*), *pressing forwards* (*Effort* and *Space*), and *sinking* combined with *enclosing* (*Shape*). Photo published with permission of a volunteer.

Five first-year students of the DMT program who did not participate in the present study and were not yet familiar with LMA, were taught and then executed these three movement sequences. Each movement sequence was repeated for 5 min. Based on self-reports of the students, we decided to use the movement sequence for condition A and B in which happiness was most recognized and experienced (“Happy” sequence). These students reported a decrease of happiness in the movement sequence that consisted of LMA movement elements that were *not* associated with the expression or experience of happiness, which made the sequence useful for condition C (“not-Happy” sequence; see excerpts from both movement sequences in Figures 1, 2). A video clip was made of both movement sequences for study purposes. Both video clips and a verbal description of each movement sequence including the LMA movement elements can be made available on request to the corresponding author.

#### DMTh and Research Assistants

In all three study conditions, the dance movement intervention was guided and/or co-executed by a DMTh or research assistant (RA). Inclusion criteria for the DMTh participating in the research experiment were (1) being a qualified DMT (bachelor or master level) and (2) experiential knowledge of LMA. RAs were DMT students (2nd and 4th year) who voluntarily assisted during this study. All received a certificate of participation. A 2-h training session for the DMTh and RA was offered by the first author prior to the experiment. The selected LMA movement elements were embodied and observation of these elements were practiced in this training, the two selected movement sequences for condition A/B and C (see “Development of the movement sequences”) were taught and the DMT techniques (attuning, mirroring) were practiced and repeated. To influence the mood of the participants as little as possible during the research conditions, all DMTh and RA were asked to be as objective, neutral, and non-suggestive as possible in their professional attitude and not to make any therapeutic contact with the participants, for example by inquiring about feelings. The DMTh and RA were given details of the research protocol in which the procedure for each condition was shown, including the link to the videos of both movement sequences and further instructions for assisting in collecting the questionnaires from the participants. The training session was completed when the first author determined that there was agreement on the observation of the selected LMA movement elements, the DMT techniques were performed and the movement sequences were memorized.

#### Pre-session and Study Experiment

The study took place in two sessions (pre-session and study experiment) in a dance studio at the university, 7 days apart. Both had a duration of 1 h. The aim of the pre-session was to familiarize participants with the LMA movement elements and the independent performance of the “Happy” movement sequence. These movements were not verbally labeled with emotions. During the pre-session, participants adequately learned the “Happy” movement sequence associated with the expression and experience of happiness, which was later tested in conditions A and B. This was instructed by the first author, who is also an experienced teacher, DMTh, and CLMA. The pre-session was completed when the first author determined that the intended LMA movement elements were adequately embodied and learned by the participants and they could perform the movement sequence independently. It was deliberately chosen to only teach one movement sequence, so the participants did not get a hint of the study purpose. Participants were asked to remember and practice the movement sequence in the meantime. For support, the participants received a link to the video clip of this movement sequence.

The second part of the study, the study experiment, took place in four separate rooms that are usually occupied for arts therapies classes at the university. Each study condition (A, B, and C) was executed in a separate room. This was done for practical reasons, so that three participants could participate in a different study condition at the same time. In the room, only a chair and a table with laptop for taking the questionnaires were present. The participants were welcomed by assisting students in another room so as not to disturb any participants performing their study conditions. Here, the participants were randomly assigned to the six different orders in which the three conditions could be performed. They received a sticker they could put on their clothing, which showed their participant number and the order they were going through all three conditions. This separate room was also used during the study to create a break in between conditions, before going to the next one.

In each condition there was either a RA (conditions B and C) or DMTh (condition A) present. Each condition lasted 10 min, of which 5 min was for the movement intervention and 5 min in total for completing the pre- and post-measurement. Between conditions, there was a break (10 min) for the participant in a separate, neutral room, after which s/he moved on to the next condition or completed the experiment. All conditions were performed without music.

All three study conditions (A, B, and C) consisted of a task for carrying out a dance movement intervention based on a specific movement sequence. During condition A, this movement sequence was repeated by the participant within the given time, during which the DMTh moved in response with the same mode of emotional expression and movement elements of the participant. During condition B, the participant could watch and follow this movement sequence shown in a video clip, on a TV screen present in the room. They were also allowed to move in their own timing. The RA mentioned beforehand that she/he would dance the sequence along with the participant to convey an atmosphere of support, but remained out of sight (position diagonally back, front facing the screen), so there was no direct eye contact or interaction during movement. During condition C, the participant was first taught the movement sequence that consisted of LMA movement elements that are not associated with the expression and experience of happiness. This was taught by a RA who was present, by means of a demonstration and movement instructions for the execution of the movement sequence. The participant was then given the task to independently repeat this movement sequence within the given time. The RA did not participate in this, but remained present in the room.

Correct execution of the protocol for each condition per participant was questioned by means of a short checklist for the RA and the DMTh. Here, RA and DMTh post checked for the execution of the LMA movement elements within the movement sequence by the participant, the use of DMT techniques (in condition A) by the DMTh and other particulars could be noted. All participants were videotaped during execution of the movement sequences for post-validation of the correct execution of the LMA movement elements when the checklist gave rise to this. Before and after the movement intervention in each condition, participants scored their affective state/mood in the current moment and physical fatigue (only post-intervention). After completing all three conditions, the participants returned to the neutral room where two RA were present. They filled in a final questionnaire which contained questions about their characteristics and their subjective experience regarding the experience of happiness during all conditions.

### Measurements

#### Demographic Variables

Demographic data were collected, including age (in years), nationality (Dutch or option “different” with the option to fill this in) and gender (male/female).

#### Positive Affect

Positive affect was measured using the PANAS (Positive and Negative Affect Schedule; Crawford and Henry, 2004). The validated Dutch version of the PANAS was used in this study (Peeters et al., 1996). The PANAS questionnaire contains 20 items that refer to a certain emotion or feeling; 10 items gage positive affect and 10 items negative affect. Happiness as a positive emotion was measured in this study by the 10 items that refer to positive affect (PANAS-p), namely: interested, cheerful, strong, enthusiastic, self-confident, proud, alert, inspired, determined, and attentive and active. Participants indicated on a five-point Likert scale to what extent they experienced a certain emotion or feeling (1 = hardly or not at all, 5 = strongly). They were asked to describe this in the current moment, in order to determine the direct effect of the intervention. A higher sum score (minimum 10, maximum 50) indicates a more positive affect. The PANAS-p subscale has high reliability, with an alpha between 0.86 and 0.90 (Watson et al., 1988). Also, the PANAS is frequently used in related studies on affect.

#### Happiness and Other Emotions

We measured happiness by using digital visual analog scales (VAS), by placing a perpendicular line on a horizontal line (100 mm). On the left side is the minimum score (“don’t feel the emotion at all” = 0), on the right is the maximum score (“feel the emotion really strongly” = 100). In addition to happiness, the participant also rated other basic emotions (angry, scared, and sad) on a VAS to disguise the emotion we were interested in. The score on the VAS is the number of millimeters between the minimum score and the line indicated by the participant. A high score on the VAS means that the emotion is experienced to a high degree. Acceptable psychometric properties have been reported for a digital VAS for measuring anxiety (Rossi and Pourtois, 2012).

#### Additional Questions on Experiences During the Movement Sequences

After completion of the whole experiment participants were asked to mark the condition where s/he experienced happiness the most (condition A, B, or C) and to nuance his/her choice for this condition by selecting from the elevens items that caused this experience. Items were, for example, “*I was able to make eye contact*,” “*Here I felt most at ease*” or “*By moving together I felt this emotion more strongly*.” Checking multiple answers was possible. In order to detect the participants’ experience with regard to movements that caused happiness in their experiences, for the item “*The type of movements made me happier*,” we added a qualitative question where the participant was asked to define these movements in their own words. The complete questionnaire (Dutch version) can be made available on request to the corresponding author.

To verify whether participants had expended approximately the same amount of physical effort in each condition and to check for fatigue effects among the participants, the amount of physical effort of each condition was rated by the participant on a VAS (100 mm) during post-measurement.

All measurements were entered digitally on a laptop that was present in the room where the movement intervention took place for conditions A, B, and C, so the experience of the participant before and after the movement intervention could be measured directly and be least affected by the environment. The RA or DMTh present asked the participants to fill in the questionnaires. The concluding questionnaire, including demographic data, was conducted in the same room where the participants were welcomed and had their break in between switching to the next condition.

### Data Analysis

First, we examined the forms that were filled in by the RA and DMTh to look for deviations from the study protocol. Second, to test whether there were differences between pre- and post-test scores per condition, we performed paired sample *t*-tests for the PANAS-p and the VAS scores. Next, to test whether the participants showed differences regarding the development of positive affect or feelings of happiness (PANAS-p and VAS) during the performance of the three conditions, we performed GLM repeated measure analyses using a 3 × 2 model (condition by time). If the assumption of sphericity was not assumed, results of the Greenhouse–Geisser test were used. Within-subject contrasts were performed when the interaction effect was significant. All the analyses were performed for the PANAS-p and the VAS separately. In addition, we tested the amount of physical effort between the three conditions using GLM repeated measure analyses with one factor (condition). Finally, answers to the questions on experiences during the movement sequences were analyzed using descriptive statistics and categorization of the answers on the open questions. All analyses were performed using IBM SPSS Statistics 24. The statistical significance (*p*) was determined at 0.05.

## Results

Examination of the forms revealed that all participants executed the movement sequences as planned in the study protocol and showed no further deviations of the intended protocol. In one case (participant 10, condition B), there was a remark that the RA and the participant were acquainted, which could have influenced the attitude of the RA and the participant. The results gave no rise to a post-session validation of each video to assess the inclusion of the LMA movement elements and no participants were excluded from analyses.

**FIGURE 3:**
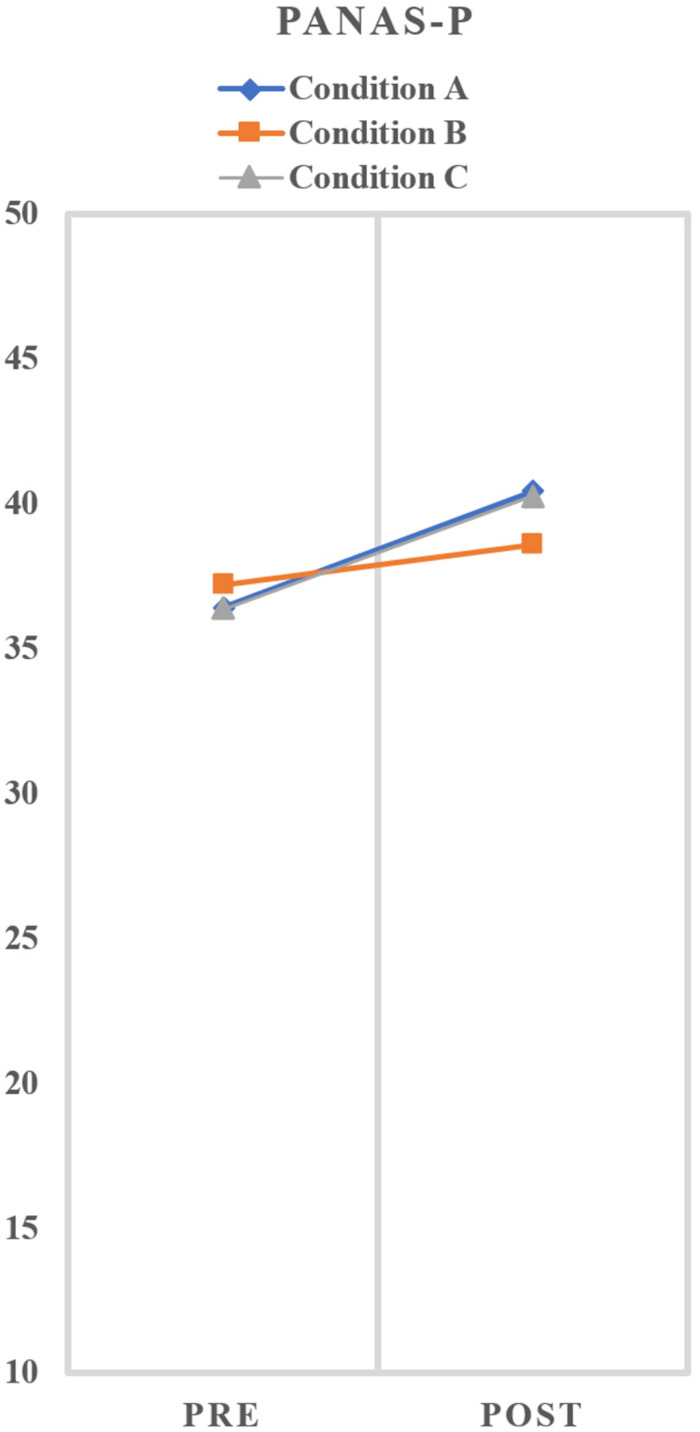
Scores PANAS-p at pre and post measurements per condition. Analysis showed a small significant interaction effect (*p* < 0.05) for condition × time.

**FIGURE 4:**
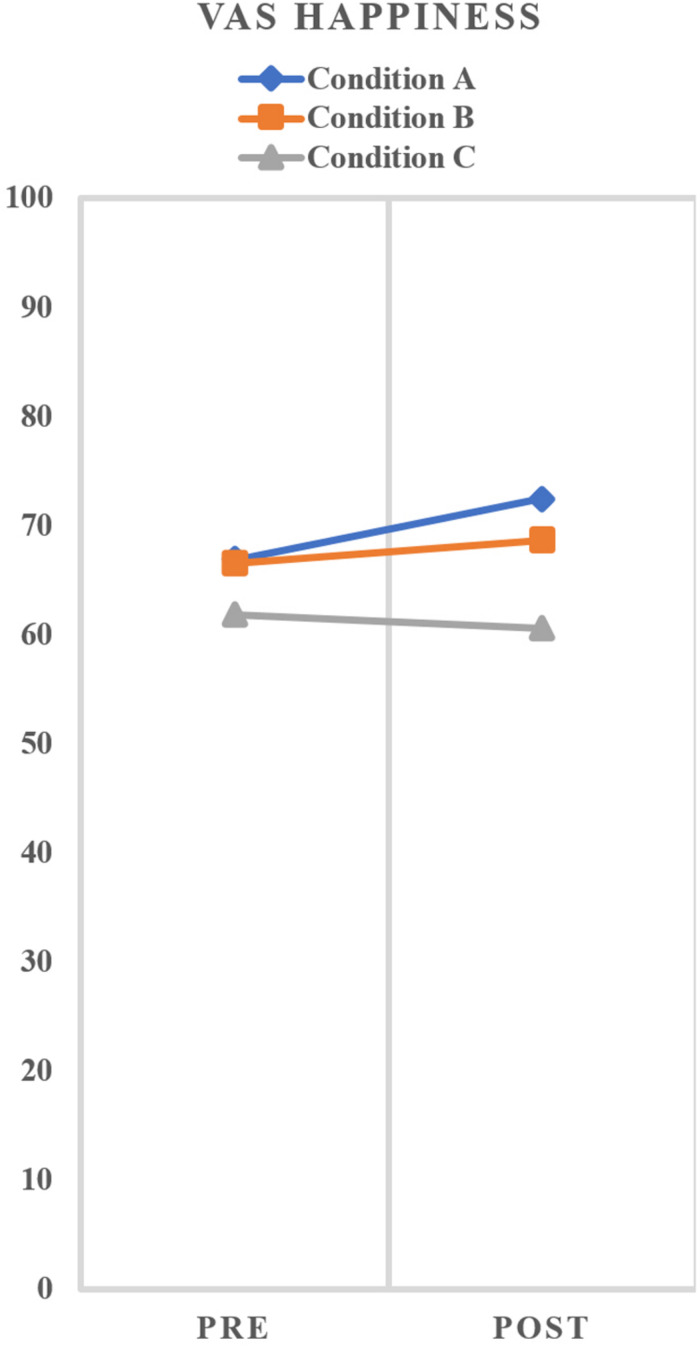
Scores VAS Happiness at pre- and post-measurement per condition. Analysis showed no significant interaction effect for condition × time.

Table 2 shows the mean and standard deviation of the scores of the PANAS-p and VAS. The paired samples *t*-test showed a significant increase of PANAS-p. We found a significant increase from pre- to post-test for condition A *t*(1,24) = −3.43, *p* < 0.01 and condition C *t*(1,24) = −5.89, *p* < 0.01, but not for condition B *t*(1,24) = −1.56, *p* < 0.13. Regarding the VAS score, we found a significant increase from pre- to post-test for condition A *t*(1,24) = −2.09, *p* < 0.05. No difference between pre- and post-test VAS score was seen for condition B *t*(1,24) = −0.70, *p* > 0.05 and condition C *t*(1,24) = 0.49, *p* > 0.05. Regarding PANAS-p, GLM repeated measures analyses showed no significant main effect for condition *F*(2,48) = 0.535, *p* > 0.05, *partial* η^2^ = 0.02. Instead, there was a significant main effect for time *F*(1,24) = 23.19, *p* > 0.05, *partial* η^2^ = 0.49 as well as a small significant interaction effect condition × time *F*(2,48) = 3.23, *p* < 0.05, *partial* η^2^ = 0.12. Figure 3 graphically shows the effects on positive affect at pre- and post-test for all three conditions. The within subject contrasts showed that there is a difference between the three conditions with respect to the increase of PANAS-p pre–post scores, in which conditions A and C both showed more increase from pre to post compared to condition B. Regarding VAS, GLM repeated measures analyses showed a significant main effect for condition *F*(2,48) = 6.36, *p* < 0.01, *partial* η^2^ = 0.21. However, there was no significant main effect for time *F*(1,24) = 1.82, *p* > 0.05, *partial* η^2^ = 0.07, nor a significant interaction effect condition × time *F*(2,48) = 1.58, *p* = 0.22, *partial* η^2^ = 0.062. Figure 4 graphically shows the effects on happiness at pre- and post-test for all three conditions.

**TABLE 2:** Mean scores (M) and standard deviation (SD) per condition (*N* = 25).

	VAS – Happiness			PANAS – *positive affect*		
	M_pre_	M_post_	M_difference_	M_pre_	M_post_	M_difference_
Condition A	66.88 (11.43)	72.48 (16.91)	5.60 (13.38)	36.44 (4.28)	40.44 (5.69)	4.00 (5.82)
Condition B	66.52 (14.30)	68.64 (15.62)	2.12 (15.24)	37.20 (4.01)	38.60 (5.89)	1.40 (4.49)
Condition C	61.80 (15.20)	60.60 (17.84)	−1.20 (12.21)	36.36 (4.40)	40.28 (3.97)	3.92 (3.33)

Results of the questionnaire on experience of the participants showed that 72% of the participants (*N* = 18) chose study condition A for having caused the strongest feeling of happiness, followed by condition C with 16% of the participants (*N* = 4) and condition B with 12% (*N* = 3). The item “*the type of movements made me happier*” was agreed by 50% of the participants for condition A, by 33.3% of the participants for condition B, and no participants rated this item for condition C. The qualitative answers of the participants that further explained this item were roughly categorized by the LMA movement elements that were selected for this movement sequence. Jumping (Body), the opening and extending of the arms (Shape) and the light, loose and free movements (Effort) were specifically mentioned.

There were differences between the conditions on the amount of physical effort *F*(2,48) = 18.174, *p* < 0.05, *partial* η^2^ = 0.431. Pairwise comparison showed that more physical effort was experienced in condition B compared to condition A [M_*dif*_ = 19.52, 95% CI (9.53, 29.51), *p* < 0.0005] and condition C [M_*dif*_ = 14.84, 95% CI (7.82, 21.86)]. There was no difference in physical effort between condition A and C [M_*dif*_ = 4.680, 95% CI (−13.51, 4.150), *p* = 0.555].

## Discussion

In this study, we investigated the effect of specific movement feedback on the experience of happiness on the individual and interpersonal levels. The findings provide evidence that the kinaesthetic movement feedback of a “happy” movement sequence while being supported by a DMTh (condition A) is best proved to increase the feeling of happiness. No significant evidence is found for the influence of dynamic body feedback on the increase of the targeted emotion when solo executing a “happy” movement sequence (condition B). The execution of movement elements not associated with happiness (condition C) had no effect on the feeling of happiness, but improved the positive affect.

The findings show improvement on both outcome measures for condition A. This condition was also identified by the majority of the participants as the condition that increases happiness the most. Here, participants were supported by a DMTh who attuned to and mirrored them during the “happy” movement sequence. Within the attuned DMT intervention, both the DMTh and the client/participant are actively involved with their own body and movement, and this ensures an interbodily play of expressions and reactions. The client’s body is informed, not only by their own body feedback, but also by perceiving the therapist’s movement expression as an additional component (Suitner et al., 2012; Fuchs and Koch, 2014). The client therefore not only experiences the emotions through body feedback and the physical resonance in his/her own body, but in addition experiences the movement qualities as reflected in the therapist’s movement. This interaction of internal sensations and external perception of the other attuning to and mirroring the personal movement patterns offers experiential deepening of kinaesthetic quality and stimulates embodied emotional awareness (Samaritter and Payne, 2016). This finding is in line with the non-verbal attunement and shared movement experiences in DMT and the effect of DMT interventions that facilitate emotion regulation and resiliency (Samaritter and Payne, 2016; Tsachor and Shafir, 2017).

Contrary to our expectations, happiness, and positive affect did not show any meaningful results for condition B on both outcome measures. These findings give no support for the association between specific movement elements associated with happiness and the elicitation of that emotion. This is in line with the inconsistency of findings on body feedback effects as demonstrated by Wagenmakers et al. (2016). In addition, condition A and C showed more similarities with the structure of the pre-session, whereas condition B not only differed in structure, but also in the presence of a RA in the back of the room. Both aspects may have affected the findings for condition B. Based on our findings for condition A, integrating an interactive dimension into the body feedback processes seems necessary to result in meaningful effects on happiness and positive affect.

Regarding the specific type of movement elements, participants frequently reported *jumping* as a movement element that contributed to their experience of happiness. This finding is in accordance with Shafir et al. (2016) who found that happiness was significantly predicted by the movement element *jumping*. In a study by Koch et al. (2007), happiness was not measured directly, but the use of rhythmic jumps in a circle dance was found to improve vitality and reduce depression. The findings of Günther (2006), in which a jump movement generated more positive feelings compared to a kick movement (which caused more negative feelings), corresponds with the participants’ reports for this item. Descriptions for the movement qualities *free flow* and *light weight* were also frequently reported to cause the experience of happiness according to the participants. These answers are in line with a study by Koch et al. (2014) in which participants reported significantly more positive memories (*p* = 0.023) and positive affect (*p* = 0.012) after moving with a light weight quality. In combination with free flow, these movement elements form a permissive way of moving with minimal (muscle) tension, which gives a cheerful, happy, and relaxed impression (Bender, 2007). *Stretching* was reported several times as an active movement element in experiencing happiness by the participants. This seems to indicate the combination of opening the torso (*spreading*) and reaching with the arms (*rising*) that were selected as movement elements for the Shape category in order to induce the experience of happiness. This effect of postural expansiveness on affect and emotional state is in line with findings (power posing makes people feel more powerful) from the study by Cuddy et al. (2018) although results must be considered with reservations due to replication studies that question this claim (Garrison et al., 2016; Cesario et al., 2017; Simmons and Simonsohn, 2017).

**FIGURE 5:**
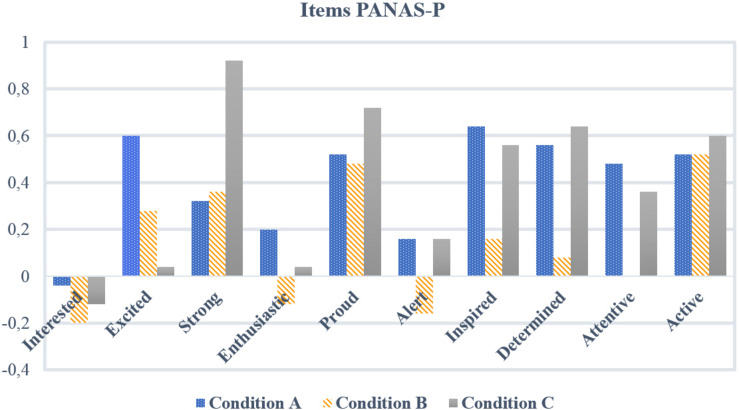
Effect PANAS-p per condition on item level.

In contrast to the effect of “happy movements,” execution of “non-happy movements” in condition C showed no effect on the feeling of happiness, but interestingly did show an increase in positive affect. This was not in line with our hypothesis nor with results in previous studies. We assumed that the selected movement elements for condition C such as *strong weight*, *direct space*, and *quick time* should rather result in the experience of negative emotions, like anger, sadness, or fear (Shafir et al., 2013, 2016). The findings also contrast with the study by Günther (2006), in which a kick movement (an effortful body action also used for the movement sequence in condition C) caused more negative feelings (i.e., more tense, fighting, aggressive feelings). An explanation for the increase in positive affect in condition C may be that the execution of these selected movement elements may also stimulate the body’s vital engagement in movement behaviors and may yield the feeling of being strong, proud, and determined. In Laban’s movement theory, some of the selected movement elements for condition C have been defined as movements with a fighting quality, which are associated with a sense of power, resistance, and strength (Laban, 1967; Bartenieff and Lewis, 1980). This line of thinking is confirmed when looking at individual items of the PANAS-p such as strong, proud, or determined. Analysis on an item level for PANAS-p showed that these specific items increased after performing the movement sequence of condition C, while this was not seen in items such as interested, excited, enthusiastic, and alert (see Figure 5). These accidental findings support the idea that in anger-inducing situations, positive affect is activated (Harmon-Jones et al., 2009). A person may also feel alert, attentive, or active in a threatening and anxious situation, or proud and strong when executing specific movement elements to express and experience a socially less accepted emotion such as anger. In additional explorative analysis of the VAS scores on “angry,” this emotion showed a significant increase for condition C. Viewed from a movement analytical perspective, anger is comparable to certain positive affects in that the movement approach related to this emotion develops from the center to the periphery (on outward or outgoing movement) and that its combination of kinetic qualities such as pressing, slashing, or punching are in LMA theory of Space Harmony in association with the three-dimensional spatial pulls. Moving with this approach and qualities in space creates a mobilizing flying and falling pattern, which can have a stimulating and regenerating impact on mood (Laban and Ullmann, 1966, 1984).

The self-report results showed that the experienced physical effort involved in the movement sequences was not the same for the three conditions. Participants reported more physical effort after performing the movement sequence in condition B, compared to conditions A and C. The experience of physical effort may have contributed to positive feelings or mood (Wood et al., 2013). The change of scores in condition B could therefore be overestimated, however, since there was no pre–post difference in condition B, this will not influence our overall conclusion. In the development of the movement sequences, we took into account a comparable physical effort per sequence, but this was not based on a solid methodology. For future studies, we recommend that the movement sequences are properly validated for the physical effort involved, before starting the experiment. Also, we suggest that objective measures for physical effort are included in addition to the subjective self-report measure.

The small sample (*N* = 25) can be seen as a limitation of this study. Therefore, replication in a larger sample would be recommended. The higher number of female participants in this study does not allow a generalization of the findings to a male population. In the same vein, results cannot be generalized to another age range. We recommend to replicate this study among both men and women above 25 years of age using a sampling strategy in the general population. Furthermore, although the combination of visual (VAS) and linguistic (PANAS) measurement instruments may be advantageous, all measuring instruments in this study were self-evaluation measurements. The results of this study could become more robust by linking these measurements to psychophysiological measurements. Further development and validation of the movement sequences can be explored in future studies, for example by comparing dynamic body feedback with more static body feedback in a control condition, or by using motion capture methods to objectively validate the movement sequences. Considering the unexpected results concerning condition B, it might be meaningful to create more equal and similar conditions in view of physical effort, structure and design of the conditions. In addition, the impact of personal movement preferences on the experience of the presented movement elements has not been explored in this study. It might be the case that not all movement elements associated with happiness need to be selected for the movement experiment to provoke the emotional experience. To further examine this assumption, we recommend to address the nature of how LMA movement elements need to be phrased and compiled in a solo movement intervention to support a shift in emotional state. A more personalized study approach might follow from this, offering participants the option to choose movement elements that would fit their individual experience of happiness. However, a personalized choice of movement elements would require thorough consideration of research methods to produce systematic and replicable results.

## Conclusion

In conclusion, this study demonstrated that happiness can be enhanced by executing a movement intervention based on LMA movement elements associated with happiness together with a DMTh, due to the resulting dynamic and interbodily feedback. The results support the role of movement in changing or influencing an emotional state and complement and expand the still limited research on the influence of dynamic body feedback on affect on the individual and interpersonal dimension. The results can be used to inform DMT-specific interventions that contribute to the regulation of emotions. More specific, this type of intervention may be well suited for young adults and perhaps be used as an effective public health intervention at schools and campuses to improve the students’ well-being. The relationship between body and emotion is an integral part of DMT, and the relationship of these two entities is the central aspect of the therapeutic process. The results of these findings indicate that dynamic movement elements are a fertile research theme with much potential for DMT practice and the opportunity for the profession to associate more with research on body feedback, the interbodily resonance that takes place between therapist and client, and the impact on the affective state.

## Data Availability Statement

The raw data supporting the conclusions of this article will be made available by the authors, without undue reservation.

## Ethics Statement

With regard to ethics approval, this study was conducted in 2017 and an ethics approval from the National Medical Ethical Board was not required as per our institution’s guidelines and national regulations (Dutch “Law of medical research involving Human Subjects” [“Wet Medisch-wetenschappelijk Onderzoek (WMO)].” The participants of this study consisted of young adults between the age of 18–25, which were asked to perform movement sequences in three conditions and fill in several questionnaires regarding their emotional state per condition before and after execution of the movement sequences. Prior to the study experiment, these participants were provided with information with regard to the research procedure and received specific movement training (1 h) to familiarize with the movements. The actual study experiment had a duration of 1 h per participant. This research procedure was not considered as a risk of bringing the participants any possible harm. All participants provided written informed consent both for the purposes of research participation as well as for the publication of the manuscript in accordance with the Declaration of Helsinki. Dancing movement sequences with specific instructions as used in this study is common in clinical practice of dance movement therapy and therefore considered as not harmful for participants. Prior to the study, the DMTh and research assistants (dance movement therapy students) who executed the research conditions were provided with written information with regard to the research aims and procedures, and participated in a 1.5 h training session. This research procedure was not considered to be at risk of bringing the dance movement therapist and DMT students any possible harm. Written informed consent was obtained from all therapists and students in accordance with the Declaration of Helsinki.

## Author Contributions

JG developed the research design, conducted the research, and first authored this manuscript. RS and SH supervised the development of the research design and co-authored this manuscript. JG and SH performed the analysis. All authors approved the manuscript and agreed to be accountable for all aspects of the work.

## Conflict of Interest

The authors declare that the research was conducted in the absence of any commercial or financial relationships that could be construed as a potential conflict of interest.
